# Immune-related lncRNA classification of head and neck squamous cell carcinoma

**DOI:** 10.1186/s12935-022-02450-z

**Published:** 2022-01-15

**Authors:** Ruoyan Cao, Lin Cui, Jiayu Zhang, Xianyue Ren, Bin Cheng, Juan Xia

**Affiliations:** 1 Hospital of Stomatology, Sun Yat-sen University, No. 56 Lingyuan Xi Road, Yuexiu District, Guangzhou, People’s Republic of China; 2 Guangdong Provincial Key Laboratory of Stomatology, Guangzhou, People’s Republic of China; 3 Guanghua School of Stomatology, Sun Yat-sen University, Guangzhou, People’s Republic of China

**Keywords:** Head and neck squamous cell carcinoma, Classification, Immune microenvironment, lncRNA, Immunotherapy

## Abstract

**Background:**

Long noncoding RNAs (lncRNAs) play a critical role in innate and adaptive immune responses. Thus, we aimed to identify ideal subtypes for head and neck squamous cell carcinoma (HNSCC) based on immune-related lncRNAs.

**Methods:**

TCGA HNSCC cohort was divided into two datasets (training and validation dataset), and 960 previously characterized immune-related lncRNAs were extracted for non-negative matrix factorization analysis. We characterized our HNSCC subtypes based on biological behaviors, immune landscape and response to immunotherapy in both training and validation cohort. A lncRNA-signature was generated to predict our HNSCC subtypes, and essential lncRNAs involved in tumor microenvironment (TME) were identified.

**Results:**

We developed and validated two HNSCC subtypes (C1 and C2) based on the 70 lncRNAs in the training and validation cohort. C2 subtype displayed good prognosis, high immune cell infiltration, immune-related genes expression and sensitivity to PD-1 blockade. C1 subtype was associated with high activity of mTORC1 signaling and glycolysis as well as high fraction of inactive immune cells. Finally, we generated a 31-lncRNA signature that could predict our above subtypes with high accurate. Additionally, TRG-AS1 was identified as the essential lncRNA involving TME formation. Knockdown of TRG-AS1 inhibited the expression of HLA-A, HLA-B, HLA-C, CXCL9, CXCL10 and CXCL11. High expression of TRG-AS1 indicated a favorable prognosis in HNSCC and anti-PD-L1 cohort (IMvigor210).

**Conclusions:**

Our study establishes a novel HNSCC classification on the basis of 31-lncRNA, helping to identify beneficiaries for anti-PD-1 treatment. In addition, a critical lncRNA TRG-AS1 is identified as a new potential prognosis biomarker as well as therapeutic target.

**Supplementary Information:**

The online version contains supplementary material available at 10.1186/s12935-022-02450-z.

## Background

Head and neck squamous cell carcinoma (HNSCC) is the sixth most common malignant tumor, with approximately 640,000 new cases worldwide each year [1]. Despite significant advancements in treatment, mortality rates for HNSCC remain at around 50%. Thus, it is essential to explore novel and effective therapeutic strategies to improve the clinical outcomes of HNSCC.

Recently, immunotherapy has received more and more attention in the area of cancer treatment owing to its remarkable and stable overall survival advantages. HNSCC might also be effective to immunotherapies since its frequent mutations and resulted neoantigens [2]. Indeed, platinum-pretreated metastatic and recurrent HNSCC receiving anti-programmed cell death (PD)-1 therapy showed durable clinical and survival benefit [3, 4]. However, the overall response rates of immunotherapy are less than 20% in unselect HNSCC patients [3, 5]. A better understanding of the tumor microenvironment (TME) formation and selection of potential beneficiaries may help increase survival benefit of immunotherapy.

Increasing evidence have proved that long noncoding RNAs (lncRNAs) played a critical role in innate and adaptive immune responses via regulating the differentiation and function of immune cells. For example, knockdown of lncRNA Pvt1 could significantly suppress the immunosuppressive function of granulocytic myeloid-derived suppressor cells [6]. In terms of adaptive immune responses, lncRNA NKILA and EGFR could reduce cytotoxic T lymphocytes (CTLs) infiltration and CTLs activity [7, 8]. The function of few lncRNAs in HNSCC immunology has been demonstrated [9, 10], however, a large number of immune-related lncRNAs have not been investigated thoroughly.

In this study, we systematically analyzed the immune-related lncRNAs in HNSCC. Two HNSCC subtypes (C1 and C2) based on the prognostic value of immune-related lncRNAs were identified in both training and validation cohort. C2 subtype exhibited higher immune cell infiltration, fractions of active immune cells, expression of immune-associated molecular and the response rate of anti-PD-1 treatment than C1 subtype in both training and validation cohort. In addition, TRG-AS1 acted an important role in regulating TME of HNSCC and might be a potential therapeutic target.

## Methods

### Data source

Level 3 RNA-Seq data consisting of 502 HNSC tissues and 44 normal controls were downloaded using TCGAbiolinks R package [11] (up to April 21, 2020). We also obtained corresponding clinical information, including age, gender, tumor grade, TNM stage, survival time, and survival status. After filtering out non-primary tumors and following up for less than 30 days, 490 HNSC samples were finally included and randomly split into two cohorts: training cohort (n = 343) and validation cohort (n = 147). The baseline information was presented in Table 1.

**Table 1:** Clinicopathological characteristics of training and validation cohort

Variable	Training cohort	Validation cohort
Age		
< 60	153 (44.61%)	65 (44.22%)
>=60	190 (55.39%)	82 (55.78%)
Gender		
Female	94 (27.41%)	35 (23.81%)
Male	249 (72.59%)	112 (76.19%)
Race		
White	291 (84.84%)	129 (87.76%)
Others	42 (12.24%)	14 (9.52%)
Unknown	10 (2.92%)	4 (2.72%)
Alcohol consumption		
No	103 (30.03%)	48 (32.65%)
Yes	235 (68.51%)	93 (63.27%)
Unknown	5 (1.46%)	6 (4.08%)
Histologic grade		
G1+G2	255 (74.34%)	97 (65.99%)
G3+G4	79 (23.03%)	40 (27.21%)
Unknown	9 (2.62%)	10 (6.80%)
Stage		
Stage I+II	61 (17.78%)	33 (22.45%)
Stage III +IV	233 (67.93%)	96 (65.31%)
Unknown	49 (14.29%)	18 (12.24%)
Vital status		
Alive	207 (60.35%)	94 (63.95%)
Dead	136 (39.65%)	53 (36.05%)

### Identification of HNSCC subtypes

A total of 960 immune-related lncRNAs were achieved from a previous study [12]. First, univariate Cox analysis was utilized to filter out lncRNAs without prognosis value (*P* < 0.05). Subsequently, non-negative matrix factorization (NMF) clustering method was employed in the training dataset using CancerSutypes R package [13]. At last, the same candidate lncRNAs were also utilized in the process of NMF clustering in the validation dataset to further verify the robustness of the above classifier.

### Function enrichment and gene set variation analysis

We applied limma R package [14] to identify differentially expressed mRNAs (DEmRNAs) based on the cut-off criteria: absolute log_2_fold change (FC) ≥ 1 and adjusted P value < 0.05. Subsequently, gene ontology (GO) and the Kyoto Encyclopedia of Genes and Genomes (KEGG) analyses were performed using clusterProfiler R package [15]. Adjusted P < 0.05 was considered statistically significant. In addition, ‘Hallmark’ gene sets were downloaded using msigdbr R package for running gene set variation analysis (GSVA) [16], which is a commonly performed method for assessing the variation in biological process activity and pathway in the expression datasets samples. Statistically significance was defined as |log_2_FC| > 0.1 and adjusted P < 0.05.

### Estimation of immune infiltration

A total of 23 types of immune cells signatures were extracted from a published study [17] and the immune cell infiltration was quantified by single-sample gene set enrichment analysis (ssGSEA) based on GSVA R package. The abundance of immune cell was verified by the MCP counter and CIBERSORT, which were obtained from TIMER website (http://timer.cistrome.org/) [18]. In addition, the stromal scores and immune scores was also calculated by ESTIMATE algorithm [19].

### Immunotherapy response and subtype prediction

To indirectly predict the immunotherapy response of our subtypes, we measured the similarity of gene expression profiles between our subclasses and immunotherapy-treated melanoma patients based on subclass mapping [20, 21]. In addition, in order to facilitate clinical application, logistic least absolute shrinkage and selector operation (LASSO) algorithm [22] was employed to predict our subtypes classification.

### Identification of critical lncRNAs involved in TME formation

Random forest R package [23] was utilized to rank the importance of lncRNAs filtered in LASSO. The correlation between the important lncRNAs and immune infiltration was evaluated.

### Cell culture

The human HNSCC cell line CAL27 was obtained from ATCC (Manassas, VA, USA). Cells were cultured in Dulbecco’s Modified Eagle’s Medium (Gibco, USA) supplemented with 10% fetal bovine serum (Gibco, USA) at 37 °C in a humidified 5% CO_2_ incubator.

### Cell transfection

A small interfering RNA (siRNA) targeting TRG-AS1 (si-TRG-AS1) and a negative control siRNA (si-NC) were designed and purchased from GenePharma (Suzhou, China). The sequence of si-TRG-AS1#1 was 5′- GGAGCUGGACUACAGUGAUdTdT-3′, and the sequence of si-TRG-AS1#2 was 5′-GCAACUACCUCAUAGAUUUdTdT-3′. Lipofectamine 3000 (Invitrogen, Carlsbad, CA, United States) was used to transfect si- TRG-AS1 and si-NC into HNSCC cells following the instructions of the manufacturer.

### Quantitative real-time PCR

Total RNA was isolated from CAL27 cells by the RNA-quick purification kit (ESscience Biotech, China) following the manufacturer’s instructions. A total of 2 µg RNA was synthesized from cDNA using HiScript III-RT SuperMix (Vazyme, China). The obtained cDNA product was used for real-time quantification PCR (RT-qPCR). The following primers were used: TRG-AS1, forward 5′-CTCCTGGCTGATCCCACT-3′, and reverse 5′-CACTATGCCATCCTGTACCAC-3′; HLA-A, forward 5′-AGATACACCTGCCATGTGCAGC-3′, and reverse 5′- GATCACAGCTCCAAGGAGAACC-3′; HLA-B, forward 5′-CTGCTGTGATGTGTAGGAGGAAG-3′, and reverse 5′ -GCTGTGAGAGACACATCAGAGC-3′; HLA-C, forward 5′-GGAGACACAGAAGTACAAGCGC-3′, and reverse 5′- ACATCCTCTGGAGGGTGTGAGA-3′; CXCL9, forward 5′-CAATTTGCCCCAAGCCCTTC-3′, and reverse 5′-TTTTCTTTTGGCTGACCTGT-3′; CXCL10, forward 5′-GGATGGCTGTCCTAGCTCTG-3′, and reverse 5′- TGAGCTAGGGAGGACAAGGA-3′; CXCL11, forward 5′- AGCCTTGGCTGTGATATTGT-3′, and reverse 5′- GGGTACATTATGGAGGCTTTCT-3′; GAPDH, forward 5′-CTCCTCCTGTTCGACAGTCAGC-3′, and reverse 5′-CCCAATACGACCAAATCCGTT-3′.

### Statistical analysis

Log-rank test was used to compare the overall survival under different conditions assessed by Kaplan Meier survival curve. The cut-off of expression was identified by survminer package. The hazard ratios (HRs) and 95% confidence intervals (CIs) of OSCC mortality risk was estimated by univariate and multivariate Cox proportional hazards models. R was used to perform all statistical analyses.

## Results

### NMF identifies two subclasses in HNSCC

**Fig. 1:**
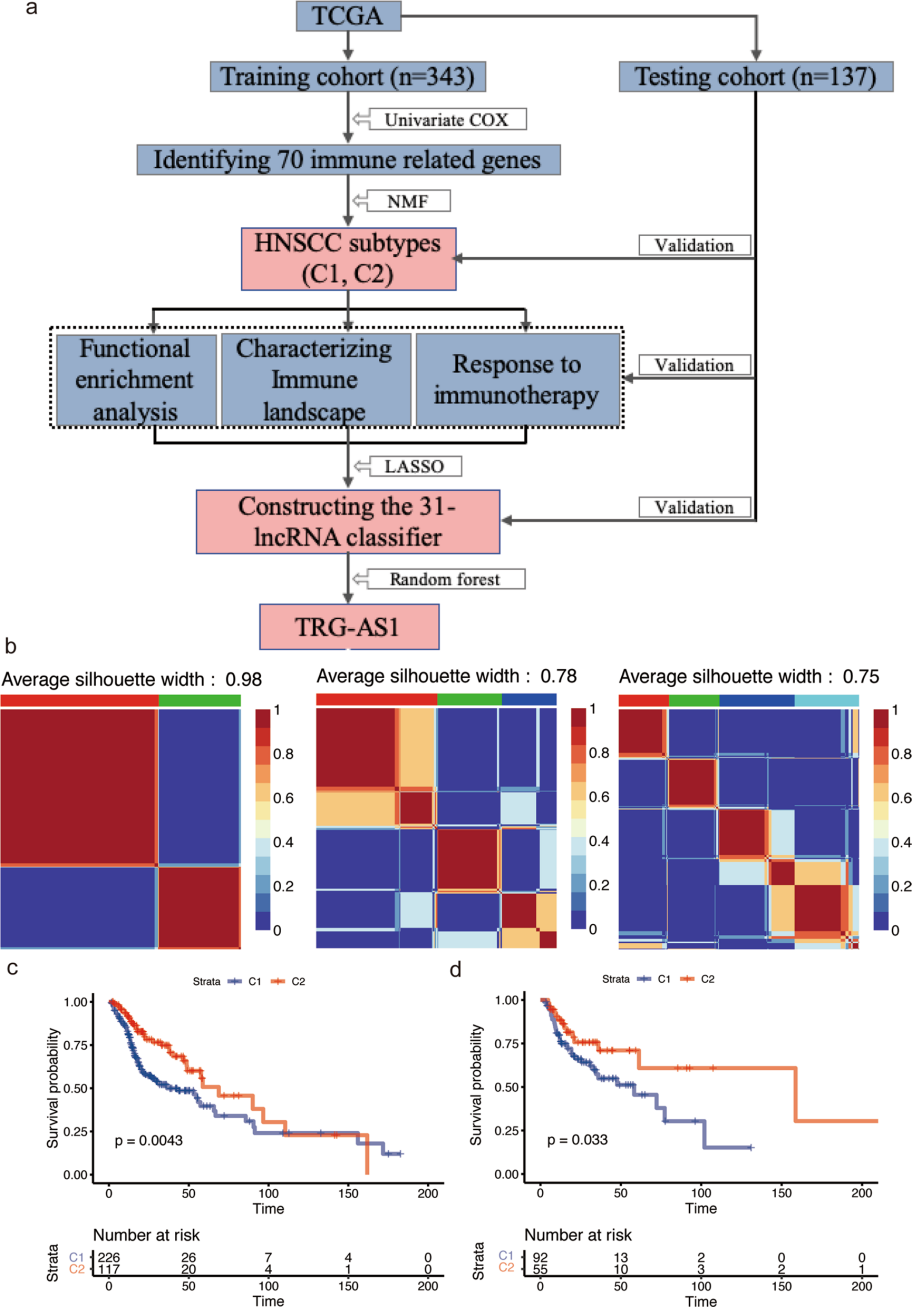
Identification of HNSCC subtype based on NMF analysis in the training cohort. **a** Flow diagram of the study. **b** NMF analysis based on 70 immune-related lncRNAs. **c**, **d** Survival analysis of the two HNSCC subtypes in training and validation cohort

A flowchart was developed to summarize our study (Fig. 1a). We obtained 70 prognosis-related candidate lncRNAs in training cohort by univariate Cox analysis, and according to their expression profile, we clustered the 343 HNSCC samples in the training cohort using NMF clustering. The results indicated that, when subtypes = 2, the maximum value of the average silhouette width (ASW) could be reached and the heatmap also maintained a highest level of similarity within a cluster, consequently, the training set should be divided into 2 subtypes (Fig. 1b). Subsequently, we applied NMF to validation cohort with 147 samples and the results also indicated that there were two distinct molecular subtypes of HNSCC (Additional file 1: Fig. S1). Based on above classification, C2 had better prognosis than C1 in training and validation cohort (Fig. 1c, d). After adjust potential confounds, C2 subtype still showed lower mortality risk compared with C1 subtype (0.52 [0.33, 0.80] in the training cohort; 0,49 [0.25, 0.94] in the validation cohort) (Table 2).

**Table 2:** Relationship between HNSCC subtypes and overall survival in different models

	Training cohort			Validation cohort		
	HR	95% CI	P value	HR	95% CI	P value
Crude	0.57	(0.39, 0.84)	0.0048	0.51	(0.27, 0.96)	0.0363
Model I	0.55	(0.37, 0.81)	0.0028	0.48	(0.25, 0.91)	0.0241
Module II	0.52	(0.33, 0.80)	0.0032	0.49	(0.25, 0.94)	0.033

Model I adjusted for age, gender, race and alcohol consumption;

Model II adjusted for age, gender, race, alcohol consumption, histologic grade and stage

### The biological behaviors in distinct lncRNA-related patterns

**Fig. 2:**
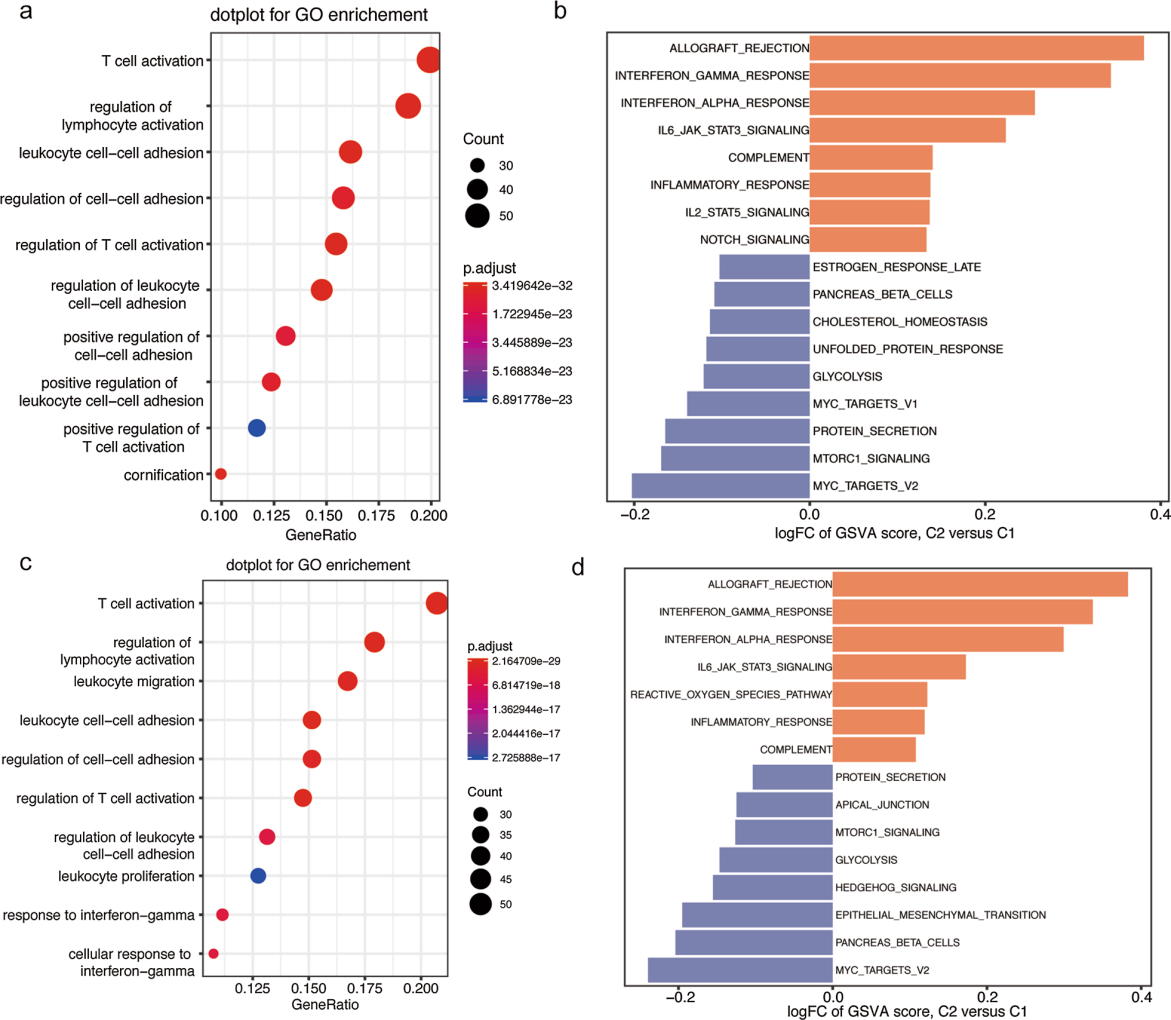
Biological characteristics of HNSCC subtypes. **a**, **c** GO analysis in training and validation cohort. **b**, **d** GSVA analysis in training and validation cohort

To better understand the biological behaviors between two subtypes of HNSCC, we first performed differential analyses. A total of 309 DEmRNAs were obtained between C1 and C2 group and these genes were mainly enriched in immune related biological processes, such as T cell activation, regulation of lymphocyte/T cell activation and cell-cell adhesion (Fig. 2a). Besides, GSVA was applied to identify different pathways between C1 and C2 group. The results showed that immune related pathways (Interferon alpha/gamma response, IL6 JAK STAT3 signaling, Complement, inflammatory response and IL2 STAT5 signaling) were activate in C2 subtype, while MYC targets, mTORC1 signaling, glycolysis and cholesterol homeostasis etc. were higher in C1 group (Fig. 2b). Validation cohort also found similar results, and thus further verified the robustness of HNSCC classification (Fig. 2c, d).

### TME cell infiltration characteristics in distinct lncRNA-related patterns

**Fig. 3:**
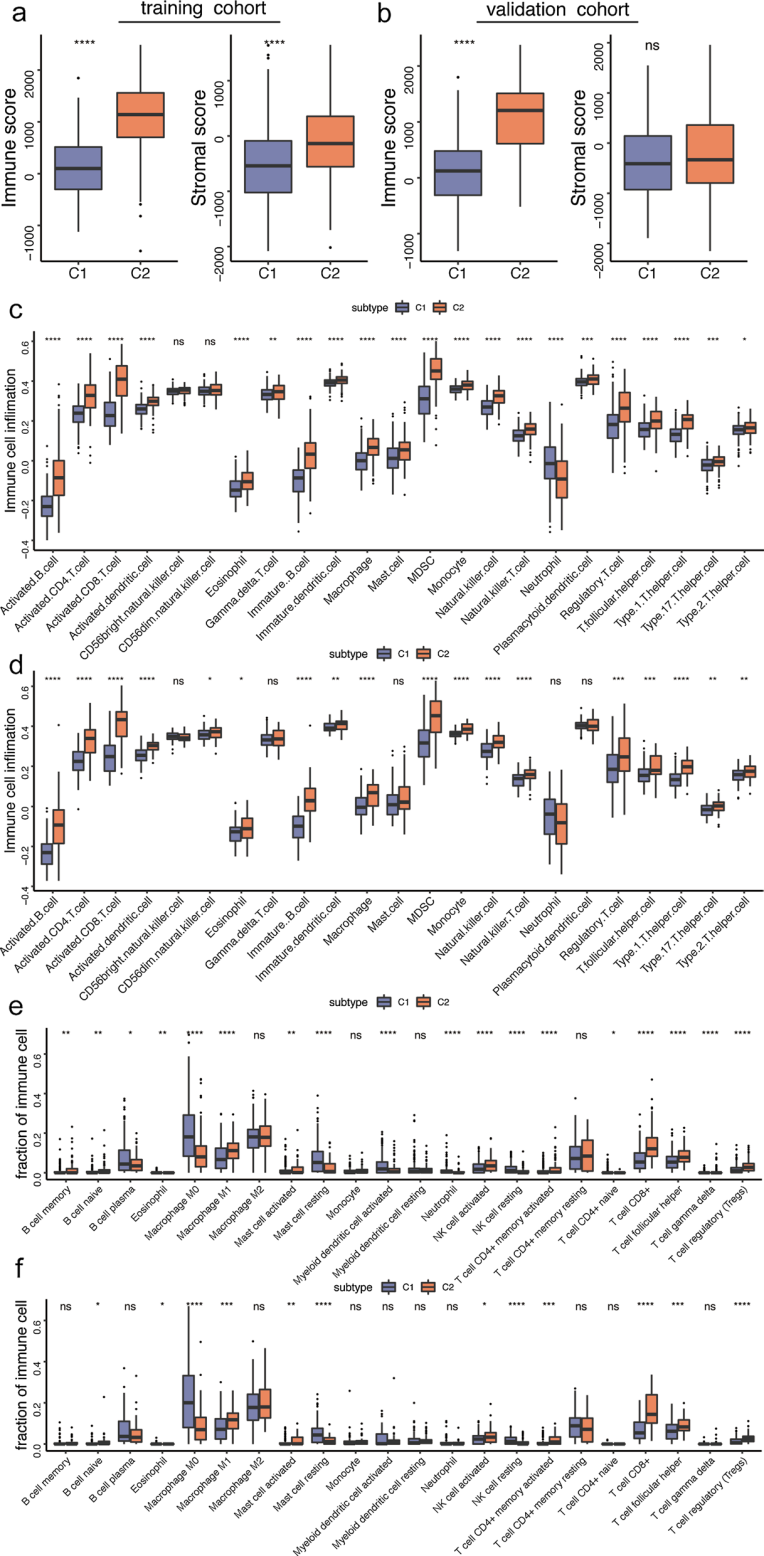
Immune characteristics of HNSCC subtypes. **a**, **b** Boxplot of immune score in training cohort and validation cohort. **c**, **d** Boxplot of immune cell infiltration in training cohort and validation cohort. **e**, **f** Boxplot of immune cell fraction in training cohort and validation cohort. (*P < 0.05, **P < 0.01, ****P < 0.0001, ns represents no significance)

Since C2 subtype activated multiple immune-related pathways, we further explored the difference in TME between C1 and C2 subtype. C2 group gained more immune score and stromal score than C1 subtype in the training cohort, while a higher stromal score was not observed in the validation cohort (Fig. 3a, b). Next, immunologic landscape was characterized based on immune cell infiltration. Corresponding to the immune score, most of immune cells infiltration was higher in C2 subtype, including innate and adaptive immunity cells (Fig. 3c, d). Furthermore, MCP analysis was also performed to validate the above results. Fortunately, we obtained similar results, that is, the infiltration of B cell, T cell, CD8+ T cell, monocyte, macrophage/monocyte and myeloid dendritic cell was higher in C2 group. In addition, cytotoxicity score was also elevated in C2 group (Additional file 2: Fig. S2). Relative proportions of 22 immune cells subsets were estimated based on CIBERSORT method. In both cohorts, the infiltration fractions of native B cell, macrophage M1, activated mast cell, activated NK cell, activated memory CD4+ T cell, CD8+ T cell, T follicular helper (TFH) and regulatory T cells (Tregs) were significantly upregulated, while eosinophil, macrophage M0, resting master cells and resting NK cells were obviously downregulated in C2 subtype than C1 subtype (Fig. 3e, f). These results indicated that C2 group might be ‘hot tumor’.

**Fig. 4:**
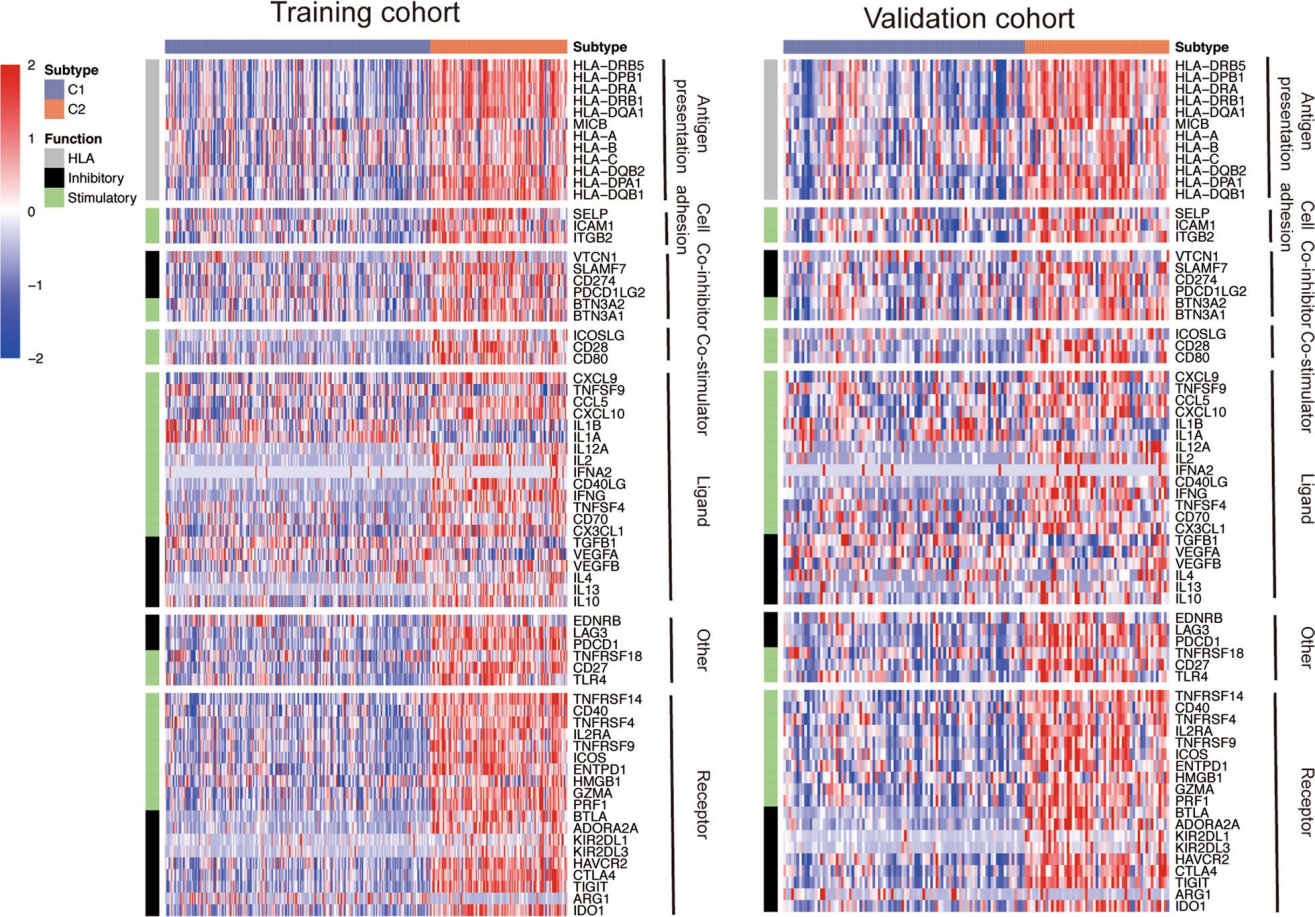
Heatmap of immune-related genes in training and validation cohort

In addition to immune infiltration, we also assessed the expression of immune-related genes between the two groups, and the results were largely consistent with immune infiltration (Fig. 4). Most HLA molecules were generally upregulated in the C2 group. Immune checkpoints, such as LAG3, PDCD1, CD274, CTLA4, and TIGIT, were observed increased in the C2 group. Moreover, we also found the expression of most immunomodulators were higher in C2 group than C1 group, including chemokines and cytolytic activity related genes, such as CXCL9, CXCL10, IL2, GZMA and RPF1. Five of the above dysregulated immune-associated genes are the member of “IFNg signature” (IDO1, CXCL10, CXCL9, HLA-DRA, STAT1 and IFNG), suggesting their positive clinical response to anti-PD-1 treatment. In summary, patients in C2 group might be more sensitive to immunotherapy.

### Distinct response to immunotherapy for lncRNA-related patterns

**Fig. 5:**
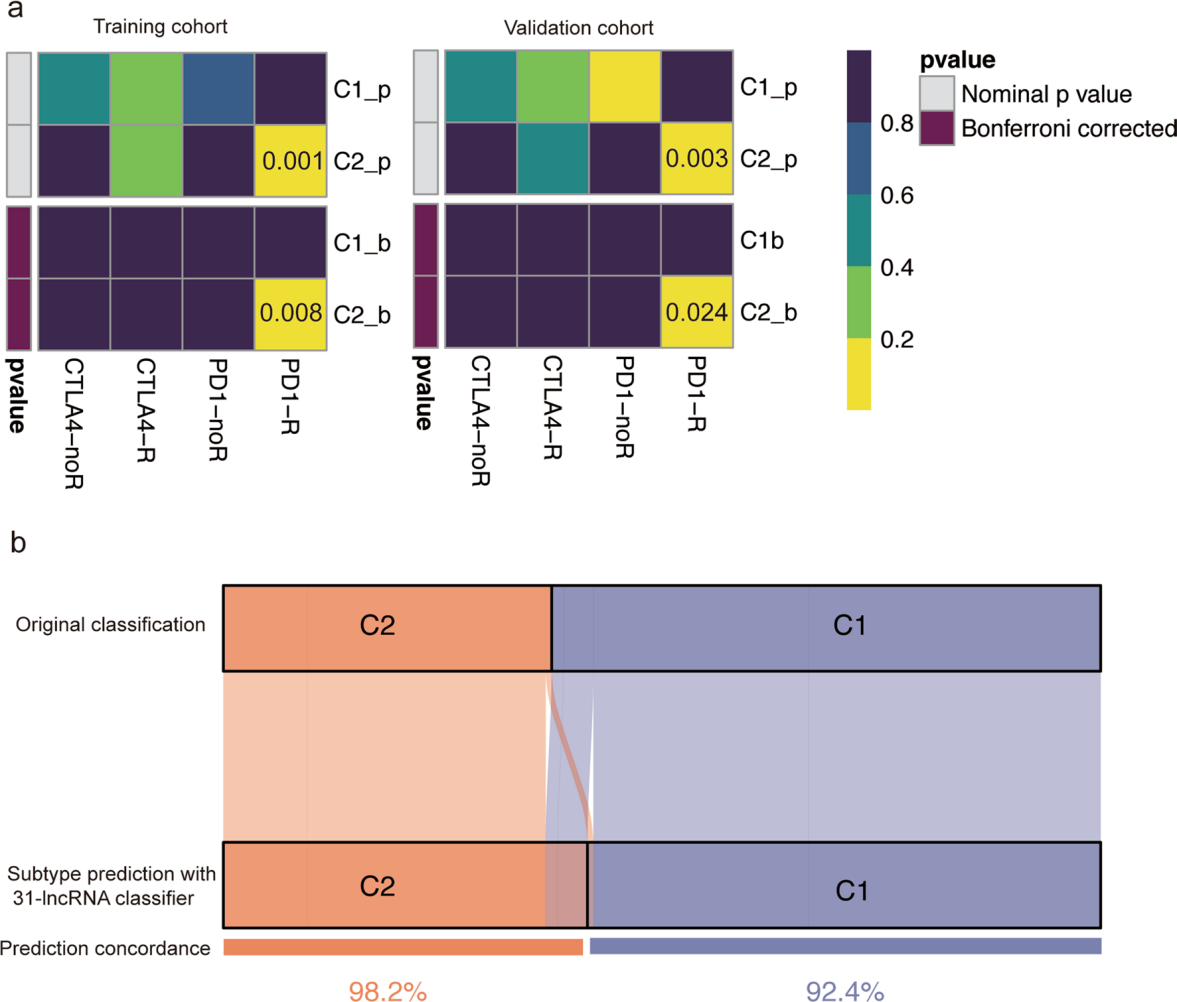
Immunotherapeutic response and identification of predictive classifier. **a** C2 may be more response to the PD-1 inhibitor (nominal and Bonferroni corrected P < 0.05) by SubMap analysis in training and validation cohort. **b** Concordance of HNSCC subtypes prediction between original classification based on NMF and the 31-lncRNA classifier

To assess the response to immunotherapy for the two subtypes, we compared their gene expression profile with a published dataset which included 47 melanoma patients who received immunotherapies. As expected, significant association was observed between the expression profile of C2 group and PD-1-response group, indicating that C2 group was more promising to respond to anti-PD-1 (Fig. 5a). Therefore, our classification of HNSCC was robustness and had potential ability to seek general susceptibilities to anti-PD-1 therapy. To better advance their clinical application, we applied LASSO to construct a prediction model in the training cohort and obtained a 31-lncRNA signature. The formula of the lncRNA-signature was shown in (Additional file 3: Table S1). Based on the cut-off value (C1: < 3.68, C2: > 3.68) of training cohort, we further explored the subtypes of validation cohort. Fortunately, we observed a high concordance (92.4% in subtype C1, 98.2% in subtype C2) in the validation cohort (Fig. 5b).

### TRG-AS1 as essential lncRNA in regulating TME

**Fig. 6:**
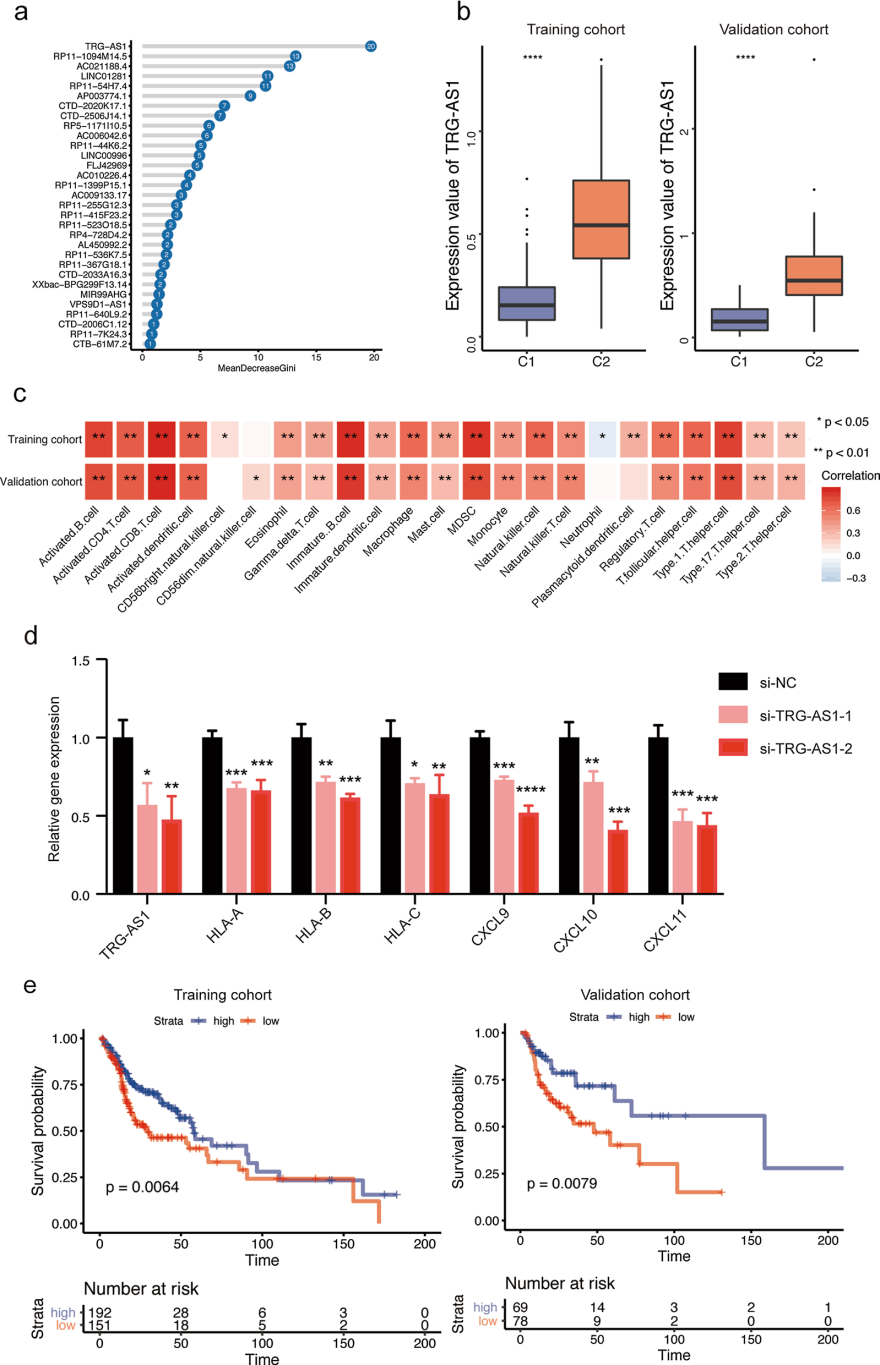
Identification of TRG-AS1 as an essential lncRNA. **a** 31-lncRNA contribution to HNSCC subtype. **b** Boxplot of TRG-AS1 expression in training cohort and validation cohort. **c** The correlation between TRG-AS1 and immune cell infiltration in training cohort and validation cohort. **d** Expression levels of TRG-AS1, HLA-A and HLA-B, HLA-C, CXCL9, CXCL10 and CXCL11 in CAL27 cells after transfection with control siRNA or TRG-AS1 siRNA. **e** Survival analysis of TRG-AS1 in training and validation cohort

To further explore the essential lncRNAs that regulated TME, the 31 lncRNAs from the above obtained lncRNA-signature were subjected to random forest analysis. The results indicated that TRG-AS1 was the most important lncRNA in our clustering (Fig. 6a). TRG-AS1 was highly expression in C2 group and it was significantly positively correlated with most types of immune cell infiltration (Fig. 6b, c). The MHC-I molecules and chemokines (e.g. CXCL9/10/11) paly an important role in immune cell infiltration, and thus we further assessed the relationship between TRG-AS1 and above genes. The results indicated that knockdown of TRG-AS1 inhibited the expression of HLA-A, HLA-B, HLA-C, CXCL9, CXCL10 and CXCL11 (Fig. 6d). As expected, the high expression of TRG-AS1 indicated a favor prognosis (Fig. 6e). Thus, TRG-AS1 might play an essential role in the TME formation of HNSCC. In addition, we also found a strong positively relationship between TRG-AS1 and immune cells in other types of tumors, especially BRCA and CHOL (Additional file 4: Table S2), which was obtained from a previous study. Moreover, OS benefit was also observed in the high TRG-AS1 expression group in the anti PD-L1 treatment cohort (IMvigor210) (Additional file 5: Fig. S3). Collectively, TRG-AS1 might be of great importance in the formation of TME and tumor development.

## Discussion

Immunotherapy is a promising treatment because it shows significant and durable clinical benefits in advanced HNSCC patients, however, less than 20% HNSCC could benefit from it, which highlights the demand to identify ideal subtypes for immunotherapy in HNSC. With the deepening in the molecular mechanisms of tumor immunity, lncRNAs attracted more and more attention due to their functions of regulating innate and adaptive immune cells response. Therefore, immune-related lncRNAs may help to explore the subtype which is more sensitive to immunotherapy.

In this study, we identified two robust subtypes (C1 and C2 subtype) based on the 70 immune-related lncRNAs with distinct TME characteristics. Compared with C1 subtype, C2 subtype presented increased immune cell infiltration and HLA molecules, suggesting initial recognition by host immune system and high level of cancer antigen presentation [24]. Higher fractions of activated immune cells (e.g. macrophage M1, activated mast/NK/memory CD4+ T cell, CD8+ T cell, TFH cell), upregulated expression of chemokines and cytolytic activity related genes as well as cytotoxicity score in C2 subtype indicated an active antitumor immune response [25–27]. Therefore, C2 subtype could be characterized by immunoinflammatory phenotype (also known as ‘hot tumor’), which maybe a possible explanation for the longer survival time of C2 subtype, and was consistent with previous studies [17, 25, 28]. Accordingly, it is reasonable to find that C2 subtype might more likely to benefit from immunotherapy owing to its TME characteristics.

C1 subtype characterized by low immune cell infiltration, decreased immune-related genes and high fraction of inactive immune cells, corresponding to immune-desert phenotype. GSVA analysis found that C1 subtype had increased activity of mTORC1 signaling, which is a metabolic master regulator. Active mTORC1 leads to the elevated glycolysis [29, 30], which is in line with our results, that is C1 subtype had higher activity of both mTORC1 signaling and glycolysis. Increased glycolysis in tumor cells are associated with immunosuppressive TME. On the one hand, as a result of glucose deprivation, tumor-infiltrating lymphocytes have decreases in antitumor effector molecules production and myeloid-derived suppressor cells were recruited to the TME [31–33]. On the other hand, an acidic TME formation hampers the expression of IFNγ in cytotoxic cells (T cells and NK cells) and promotes IL-17-mediated and IL-23-mediated inflammation [34, 35]. These might partly explain the TME characteristics as well as the poor prognosis of C1 subtype. Accordingly, it is reasonable to speculate the combination of glycolysis inhibitor and immune-checkpoint inhibitor might improve clinical outcome of C1 subtype.

In addition to identify and validate robust subtypes with distinct TME characteristics in HNSCC, we still found that lncRNA TRG-AS1, T cell receptor gamma locus antisense RNA 1, might play a critical role in regulating TME and might serve as potential prognosis biomarker as well as therapeutic target. Knockdown of TRG-AS1 suppressed the expression of HLA-A, HLA-B, HLA-C, CXCL9, CXCL10 and CXCL11. HLA-A, HLA-B and HLA-C play an important role in antigen presentation, thereby initiating an immune response. The expression of CXCL9, CXCL10 and CXCL11 is positively correlated with the density of tumor infiltrating NK and T cells [36]. Thus, it was not surprising to find that TRG-AS1 was significant positively correlated with multiple immune cell infiltration and long survival time. In addition, high expression of TRG-AS1 was also correlated with more immune cell infiltration in multiple tumors as well as OS benefit in the anti PD-L1 treatment cohort, mainly including bladder and kidney cancer. Thus, the above results highlighted the necessary to explore the function of TRG-AS1 in pan-cancer.

## Conclusions

In summary, we generate a novel HNSCC classifier based on 31-lncRNA, which helps to identify ideal candidates for anti-PD-1 treatment. In addition, an important lncRNA TRG-AS1 is identified to a novel potential prognosis biomarker as well as therapeutic target.

## Supplementary Information

**Additional file 1: Fig. S1.** Validating the HNSCC subtypes in validation cohort.

**Additional file 2: Fig. S2.** Boxplot of Immune cell infiltration based on MCP method in training cohort and validation cohort.

**Additional file 3: Table S1.** The formula of the 31-lncRNA signature.

**Additional file 4: Table S2.** The correlation between TRG-AS1 and immune cell infiltration in multiple tumor.

**Additional file 5: Fig. S3.** Survival analysis of TRG-AS1 in the anti PD-L1 treatment cohort (IMvigor210).

## Data Availability

The datasets used during the current study are available from the Cancer Genome Atlas (TCGA) dataset (https://portal.gdc.cancer.gov/).

## Abbreviations

lncRNAs: Long noncoding RNAs
HNSCC: Head and neck squamous cell carcinoma
TME: Tumor microenvironment
PD: Programmed cell death
CTLs: Cytotoxic T lymphocytes
NMF: Non-negative matrix factorization
DEmRNAs: Differentially expressed mRNAs
KEGG: Kyoto Encyclopedia of Genes and Genomes
GSVA: Gene set variation analysis
ssGSEA: Single-sample gene set enrichment analysis
LASSO: Logistic least absolute shrinkage and selector operation
WGCNA: Weighted gene co-expression network analysis
HRs: Hazard ratios
ASW: Average silhouette width
TFH: Follicular helper
Tregs: Regulatory T cells
