# Supplementary figures and images for: Immune-related lncRNA classification of head and neck squamous cell carcinoma
Source: Cancer Cell Int. 2022 Jan 15;22:25. doi: 10.1186/s12935-022-02450-z (PMC8760760; doi:10.1186/s12935-022-02450-z)

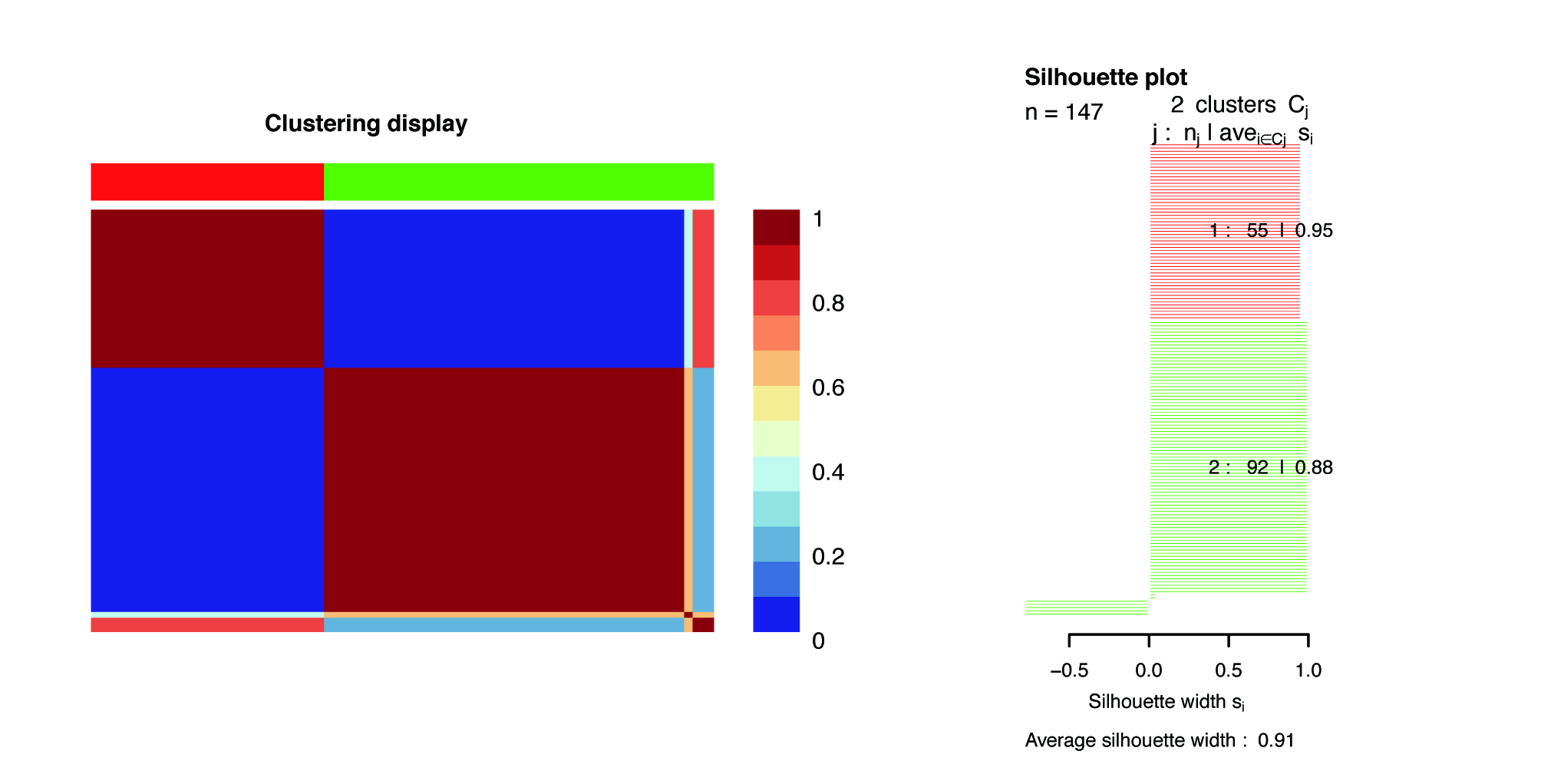

Supplement: Supplementary file 1 — Additional file 1: Fig. S1. Validating the HNSCC subtypes in validation cohort. [file 12935_2022_2450_MOESM1_ESM.tif]

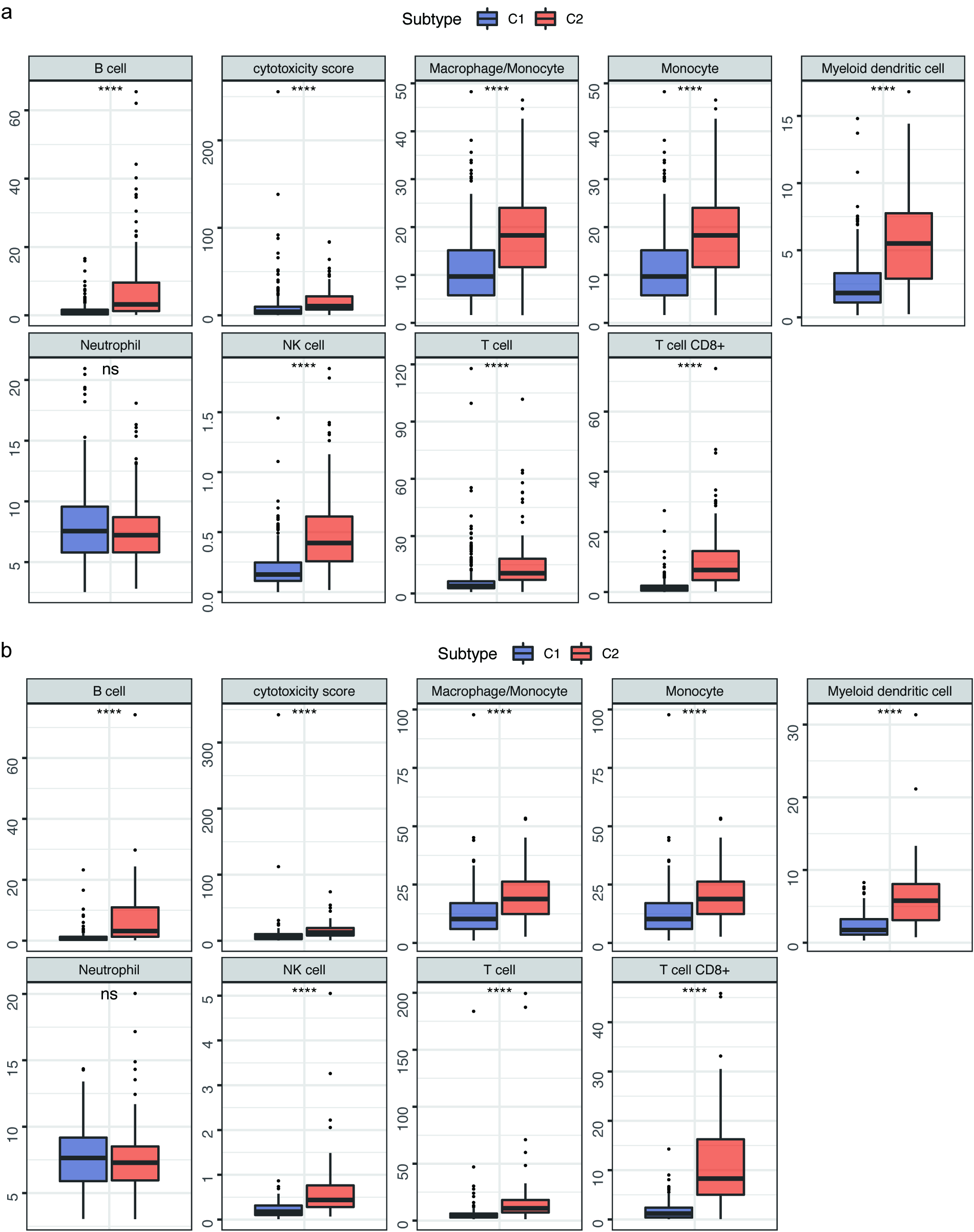

Supplement: Supplementary file 2 — Additional file 2: Fig. S2. Boxplot of Immune cell infiltration based on MCP method in training cohort and validation cohort. [file 12935_2022_2450_MOESM2_ESM.tif]

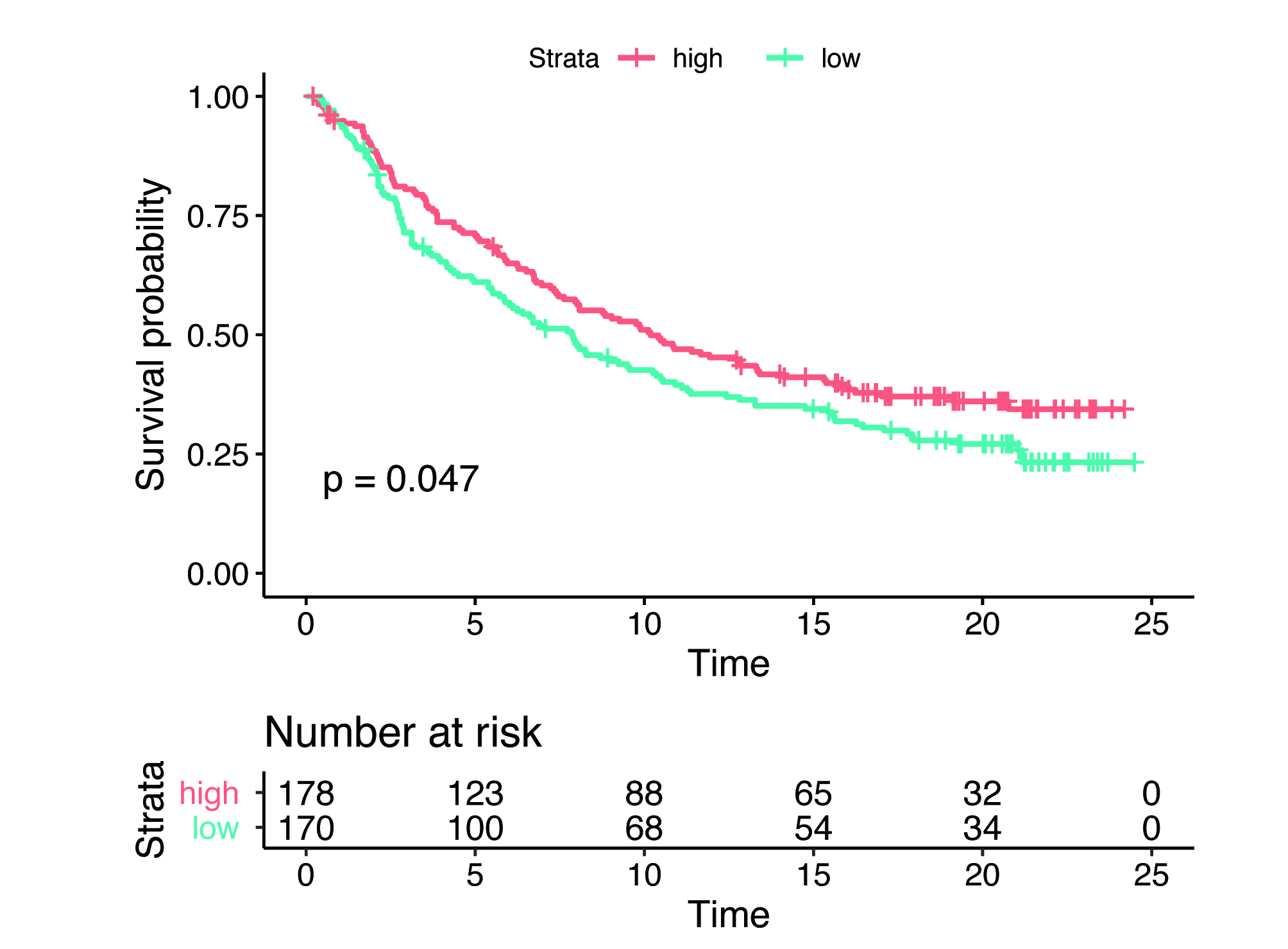

Supplement: Supplementary file 5 — Additional file 5: Fig. S3. Survival analysis of TRG-AS1 in the anti PD-L1 treatment cohort (IMvigor210). [file 12935_2022_2450_MOESM5_ESM.tif]
