# Supplementary material for: Immune-related lncRNA classification of head and neck squamous cell carcinoma
Source: Cancer Cell Int. 2022 Jan 15;22:25. doi: 10.1186/s12935-022-02450-z (PMC8760760; doi:10.1186/s12935-022-02450-z)
Supplement: Supplementary file 3 — Additional file 3: Table S1. The formula of the 31-lncRNA signature. [file 12935_2022_2450_MOESM3_ESM.docx]

The formula of the lncRNA-signature: 0.24392* MIR99AHG + 2.05467* AC006042.6 + 1.30802* RP11-536K7.5 +3.86265*AC021188.4 - 0.11363* RP11-367G18.1 + 4.07724*RP11-523O18.5+ 0.26432* AL450992.2 + 0.8899* LINC01281 + 5.19857*AP003774.1 + 0.6158*RP4-728D4.2 + 0.6906*LINC00996 + 5.76727*CTD-2506J14.1 - 1.12219*FLJ42969 + 1.49432*AC010226.4 + 1.50508*RP11-44K6.2 - 0.59152*RP11-255G12.3 + 0.76534*AC009133.17 - 0.25405*RP11-7K24.3- 0.46379* VPS9D1-AS1 - 1.51232*CTD-2033A16.3 + 1.18235*RP5-1171I10.5 + 2.23078*RP11-1094M14.5 + 0.53354*CTD-2020K17.1+ 0.7312*CTD-2006C1.12- 0.14059*CTB-61M7.2 + 0.11732*XXbac-BPG299F13.14 + 0.83061*RP11-415F23.2 + 1.61482*RP11-1399P15.1 - 1.3064*RP11-54H7.4 + 0.7092* RP11-640L9.2 + 2.45214* TRG-AS1
